# The LISA randomized trial of a weight loss intervention in postmenopausal breast cancer

**DOI:** 10.1038/s41523-020-0149-z

**Published:** 2020-02-21

**Authors:** Pamela J. Goodwin, Roanne J. Segal, Michael Vallis, Jennifer A. Ligibel, Gregory R. Pond, André Robidoux, Brian Findlay, Julie R. Gralow, Som D. Mukherjee, Mark Levine, Kathleen I. Pritchard

**Affiliations:** 1 Department of Medicine, Division of Clinical Epidemiology at the Lunenfeld-Tanenbaum Research Institute, Mount Sinai Hospital, University of Toronto, Toronto, ON Canada; 2 Department of Internal Medicine, Division of Medical Oncology, Ottawa Hospital Regional Cancer Center, University of Ottawa, Ottawa, ON Canada; 3 Primary Care, Capital Health, Department of Family Medicine, Dalhousie University, Halifax, Nova Scotia UK; 4 Department of Medicine, Dana-Farber Cancer Institute, Harvard Medical School, Boston, MA USA; 5 Department of Oncology, Ontario Clinical Oncology Group, Juravinski Hospital and Cancer Center, McMaster University, Hamilton, ON Canada; 6 Faculty of Medicine, Department of Nutrition, Research Center, Centre Hospitalier de l’Université de Montréal, Montréal, QC Canada; 7 Department of Oncology, Niagara Health System, Walker Family Cancer Center, McMaster University, Hamilton, ON Canada; 8 Department of Medicine, Division of Oncology, University of Washington School of Medicine, Seattle, WA USA; 9 Department of Oncology, Juravinski Cancer Center, McMaster University, Hamilton, ON Canada; 10 Division of Medical Oncology & Hematology, Sunnybrook Odette Cancer Center, Ontario Clinical Oncology Group, University of Toronto, Toronto, ON Canada

**Keywords:** Breast cancer, Breast cancer

## Abstract

Obesity has been associated with poor breast cancer (BC) outcomes. We investigated whether a standardized, telephone-based weight loss lifestyle intervention in the adjuvant setting would impact BC outcomes. We conducted a multicenter trial randomizing women 1:1 to mail-based educational material alone (control) or combined with a standardized, telephone-based lifestyle intervention that focused on diet, physical activity, and behavior and involved 19 calls over 2 years to achieve up to 10% weight loss. In all, 338 (of 2150 planned) T1-3, N0-3, M0 hormone receptor positive BC patients with body mass index (BMI) ≥24 kg/m^2^ receiving adjuvant letrozole were randomized (enrolment ended due to funding loss). The primary outcome was disease-free survival (DFS); secondary outcome was Overall Survival (OS). At 8 years’ median follow-up, in a planned analysis, DFS and OS were compared using the Kaplan–Meier method. Baseline BMI and other characteristics were similar between study arms. In all, 22 of 171 (12.9%) in the lifestyle intervention arm versus 30 of 167 (18.0%) in the education had DFS events; the hazard ratio (HR) was 0.71 (95% confidence interval [CI]: 0.41–1.24, *p* = 0.23). Although loss of funding reduced sample size, we view these hypothesis generating results as compatible with our hypothesis of a potential beneficial effect of a lifestyle intervention on DFS. They provide support for completion of ongoing randomized controlled trials of the effect of lifestyle interventions in BC outcomes.

## Introduction

Obesity has been associated with poor breast cancer (BC) outcomes in numerous studies. A recent meta-analysis concluded that women with obesity have an approximately one-third increased risk of BC-related mortality and a 41% increased risk of overall mortality, compared to women who are normal weight.^1^ An association of obesity with poor BC outcomes is biologically plausible; correlates of obesity, including higher levels of circulating insulin, glucose, leptin, postmenopausal estrogen, and some inflammatory markers have been associated with poor BC outcomes.^2^ This biologic plausibility strengthens (but does not prove) the hypothesis that obesity leads to poor breast cancer outcomes. The available observational evidence also does not address the issue of whether weight loss in breast cancer survivors will lower the risk of recurrence and death; well-designed randomized trials using effective weight loss interventions will most effectively provide that information.

Previous research has demonstrated that lifestyle-based interventions can lead to weight loss in women with newly diagnosed BC.^3–5^ The major focus of the studies reported to date has been on weight loss, rather than BC outcomes. Interventions have involved reduction in caloric intake and increases in physical activity, combined with behavioral and motivational support and have been delivered in person and by mail. The design of these lifestyle interventions has evolved over time, as understanding of the most effective approaches to weight loss has evolved.^6^ The most effective current approaches are based on Social Cognitive Theory and have used the results of interventions, such as the Diabetes Prevention Program and Look Ahead (also developed and tested in diabetic subjects).^7^ It is recognized that the most effective interventions use a comprehensive approach that includes an initial intense phase (minimum 6 months, including 14 contacts) designed to lower caloric intake, enhance physical activity, and address behavioral aspects of weight loss. This intensive phase, which leads, on average, to an 8% weight loss, is followed by a less intense maintenance program (minimum 1 year) that continues the lifestyle changes adopted during the intensive phase and addresses issues relating to relapse prevention and maintenance of weight loss involving at least monthly contacts with a trained interventionist.^8^

In 2007, we commenced a Phase III randomized trial (The LISA Study) to examine the impact of a weight loss intervention with educational materials versus educational materials alone, on BC outcomes.^9^ We used a 2-year telephone-based intervention that was a modification of the Diabetes Prevention Program,^7^ the most widely accepted weight loss intervention at the time of study design. As previously reported, our original design was to accrue 2150 women, sufficient to provide 80% power to detect a HR of 0.75 for our primary endpoint, disease free survival (DFS). Funding was discontinued in December 2009 after 338 women were enrolled. The smaller sample size markedly lowered our power to identify the effect we had hypothesized, nonetheless, given the strengths of our study design and early evidence of weight loss with our lifestyle intervention, we completed the intervention in all subjects (the impact on weight, diet, physical activity and quality of life to 24 months has been previously reported).^9^ At the time of discontinuation of accrual, we planned a time dependent analysis of DFS and overall survival (OS) when all subjects had been followed for at least 8 years. We report the results of those DFS and OS analyses here.

## Results

### Study population

In all, 171 patients were randomized to the lifestyle intervention arm and 167 to the education only arm. One woman randomized to the lifestyle intervention was premenopausal and was removed from the study prior to receiving any intervention. One woman randomized to the education only arm received the lifestyle intervention. One patient in the lifestyle intervention arm had a prior kidney malignancy and withdrew prior to receiving any intervention, and a second woman randomized to the lifestyle intervention was diagnosed 7 years prior to randomization—she received the study intervention. All women were included in the intention to treat analysis and had completed a minimum of 8 years of follow-up at the time of data lock (9 May 2018).

Baseline characteristics, shown in Table 1, were well balanced between study arms. Mean age was 61.6 years in the lifestyle intervention arm and 60.4 in the education only arm. In all, 95% of women were white. Mean BMI were 31.4 and 31.1 kg/m^2^ in the lifestyle intervention and education only arms, respectively. The majority of women had T1 N0 tumors that were grade 1 or 2. All women had ER + and/or PR + BC and were on letrozole at randomization. BCs were HER2 positive in 8.8% (lifestyle intervention) and 15% (education only). Just over one-third of women had mastectomy and slightly more than half received chemotherapy. Only 5.5% of women in the lifestyle arm and 9.0% in the education only arm were current smokers.

**Table 1:** Baseline characteristics of the LISA study population—patients, tumor and treatment related characteristics in subjects in the lifestyle intervention arm and education only arm.

	Education only arm	Lifestyle intervention arm characteristic
Randomized, *N*	167	171
Stratum		
BMI (kg/m^2^)		
24–30, *N* (%)	76 (45.5)	73 (42.7)
>30, *N* (%)	91 (54.5)	98 (57.3)
Prior adjuvant chemotherapy		
Yes, *N* (%)	96 (57.5)	96 (56.1)
No, *N* (%)	71 (42.5)	75 (43.9)
Intervention language		
English, *N* (%)	147 (88.0)	151 (88.3)
French, *N* (%)	20 (12.0)	20 (11.7)
Demographics		
Age		
Mean (SD)	60.4 (7.8)	61.6 (6.7)
Race		
White, *N* (%)	162 (97.0)	161 (94.2)
Asian, *N* (%)	3 (1.8)	3 (1.8)
Black, *N* (%)	0 (0.0)	4 (2.3)
Native, *N* (%)	0 (0.0)	1 (0.6)
Other, *N* (%)	2 (1.2)	2 (1.2)
Height (cm)		
Mean (SD)	161.6 (6.2)	162.0 (6.4)
Weight (kg)		
Mean (SD)	81.0 (14.4)	82.7 (15.3)
Body mass index		
Mean (SD)	31.1 (5.3)	31.4 (5.0)
Median (range)	30.4 (24.0–55.2)	30.7 (24.0–60.7)
Smoking history		
Currently, *N* (%)	15 (9.0)	9 (5.3)
Previous (<6 months), *N* (%)	9 (5.4)	8 (4.7)
Previous (>6 months), *N* (%)	60 (35.9)	72 (42.1)
Never, *N* (%)	83 (49.7)	82 (48.0)
Marital status		
Currently married, *N* (%)	119 (71.3)	123 (71.9)
Single, *N* (%)	12 (7.2)	18 (10.5)
Widowed, *N* (%)	12 (7.2)	12 (7.0)
Divorced/separated, *N* (%)	22 (13.2)	18 (10.5)
Not given, *N* (%)	2 (1.2)	0 (0.0)
Living situation		
Spouse/partner and children, *N* (%)	24 (14.4)	27 (15.8)
Spouse/partner only, *N* (%)	91 (54.5)	91 (53.2)
Children only, *N* (%)	8 (4.8)	7 (4.1)
Other relatives, *N* (%)	5 (3.0)	7 (4.1)
Other non-relatives, *N* (%)	1 (0.6)	2 (1.2)
Alone, *N* (%)	35 (21.0)	32 (18.7)
Other, *N* (%)	3 (1.8)	5 (2.9)
Tumour characteristics		
T status		
1, *N* 9%)	103 (61.7)	114 (66.7)
2, *N* (%)	56 (33.5)	47 (27.5)
3, *N* (%)	6 (3.6)	10 (5.9)
Missing/NA	1 (0.6)	0 (0.0)
*N* status		
0, *N* (%)	106 (63.5)	107 (62.6)
1, *N* (%)	54 (32.3)	49 (28.7)
2, *N* (%)	6 (3.6)	14 (8.2)
3^a^, *N*(%)	1 (0.6)	1 (0.6)
Overall grade		
I, *N* (%)	41 (24.6)	38 (22.2)
II, *N* (%)	73 (43.7)	96 (56.1)
III, *N* (%)	51 (30.5)	37 (21.6)
Missing/NA, *N* (%)	2 (1.2)	0 (0.0)
ER status		
Positive, *N* (%)	166 (99.4)	167 (97.7)
Negative, *N* (%)	1 (0.6)	4 (2.3)
PR status		
Positive, *N* (%)	145 (86.8)	144 (84.2)
Negative, *N* (%)	21 (12.6)	27 (15.8)
Missing/NA, *N* (%)	1 (0.6)	0 (0.0)
HER2 status		
Positive, *N* (%)	25 (15.0)	15 (8.8)
Negative, *N* (%)	138 (82.6)	153 (89.5)
Missing/NA, *N* (%)	4 (2.4)	3 (1.8)
Prior treatment		
Irradiation therapy		
No, *N* (%)	42 (25.2)	33 (19.3)
Yes, *N* (%)	125 (74.9)	138 (80.7)
Surgery type		
Mastectomy, *N* (%)	64 (38.3)	60 (35.1)
Lumpectomy, *N* (%)	113 (67.7)	120 (70.2)
Axillary node dissection, *N* (%)	94 (56.3)	96 (56.1)
Sentinel node biopsy, *N* (%)	113 (67.7)	108 (63.2)
Systemic chemotherapy		
No, *N* (%)	69 (41.3)	75 (43.9)
Yes, *N* (%)	98 (58.7)	96 (56.1)
Chemo- and endocrine therapy type		
AC, *N* (%)	17 (10.2)	13 (7.6)
AC Taxol, *N* (%)	29 (17.4)	21 (12.3)
FEC 100, *N* (%)	5 (3.0)	11 (6.4)
FEC Taxotere, *N* (%)	24 (14.4)	28 (16.4)
CEF, *N* (%)	2 (1.2)	0 (0.0)
Tamoxifen, *N* (%)	14 (8.4)	11 (6.4)
Anastrozole, *N* (%)	8 (4.8)	8 (4.7)
Exemestane, *N* (%)	2 (1.2)	0 (0.0)
Herceptin, *N* (%)	24 (14.4)	12 (7.0)
Lapatinib, *N* (%)	0	0
Months from diagnosis median (range)	9.1 (2.0–37.0^b^)	9.4 (1.6–92.0^b^)
Months from definitive surgery median (range)	7.3 (1.0–34.9)	7.6 (0.2–91.6)

*BMI* body mass index, *SD* standard deviation, *NA* not available, *ER* estrogen receptor, *PR* progesterone receptor, *HER2* human epidermal growth factor receptor 2, *AC* Adriamycin-cyclophosphamide, *FEC* fluouracil-epirubicin-cyclophosphamide, *CEF* cyclophosphamide-epirubicin-fluouracil.

^a^N3 patients were included in initial protocol.

^b^One patient was diagnosed 7.7 (waiver) years prior to trial randomization. Two lifestyle intervention arm patients and two education only arm subjects were diagnosed between 36 to 39 months prior to randomization, however, surgery was <36 months prior to randomization. All other patients were diagnosed <36 months prior to randomization.

**Fig. 1:**
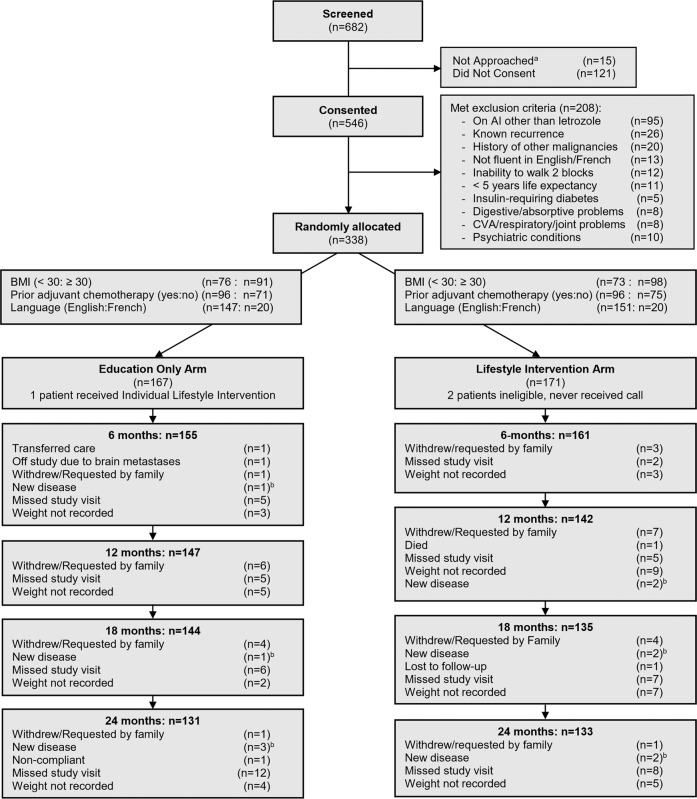
CONSORT diagram of patient flow. Summary of the recruitment and follow-up of patients enrolled onto the LISA study. *LISA* Lifestyle Study Adjuvant; *AI* aromatase inhibitor; *CVA* cerebrovascular accident; *BMI* body mass index. ^a^5 patients were not approached for the following reasons: BMI out of range (*n* = 12), on another trial (*n* = 1), blank/unknown (*n* = 2). ^b^Remained on-study for follow-up, but excluded from secondary outcomes analysis.

Just under 10% of women withdrew from the study (9.9% lifestyle intervention arm, 9.6% education only arm). Approximately 7% were lost to follow-up (6.4% lifestyle intervention arm, 7.2% education only arm). (see CONSORT diagram, Fig. 1).

### Weight loss

Overall weight loss was significantly (*p* < 0.001) greater in the lifestyle intervention arm versus education only arm (−5.3% versus −0.6% at 6 months, −5.5% versus −0.6% at 12 months, and −3.7% versus −0.4% at 24 months). This pattern was observed within each BMI (24–30 kg/m^2^ versus 30 kg/m^2^) and adjuvant chemotherapy (no versus yes) stratum. With longer follow-up, weight loss in the lifestyle intervention arm was not sustained, regardless of baseline BMI ≤ / > 30 kg/m^2^ (Table 2).

**Table 2:** Weight (measured in clinic in indoor clothing without shoes) at study entry (baseline) and change in weight during the subsequent 96 months in patients enrolled onto the lifestyle intervention arm and education only arm.

Weight (kg)	Education only arm		Lifestyle intervention arm	
	*N*	Mean (SD)	*N*	Mean (SD)
Baseline	167	81.0 (14.4)	171	82.7 (15.3)
6 months				
Absolute		80.2 (13.9)		77.6 (15.4)
	155		161	
Change from baseline		−0.5 (3.7)		−4.3 (4.1)
% Change from baseline		−0.6 (4.5)		−5.3 (4.9)
12 months				
Absolute		79.3 (14.0)		77.5 (14.9)
	147		142	
Change from baseline		−0.5 (5.3)		−4.5 (5.4)
% Change from baseline		−0.6 (6.3)		−5.5 (6.4)
18 months				
Absolute		79.0 (14.2)		78.5 (15.9)
	144		134	
Change from baseline		−0.6 (5.8)		−3.8 (5.8)
% Change from baseline		−0.8 (6.8)		−4.6 (6.9)
24 months				
Absolute		78.8 (13.2)		78.9 (15.3)
	132		135	
Change from baseline		−0.3 (5.3)		−3.1 (6.1)
% Change from baseline		−0.4 (6.4)		−3.7 (7.6)
36 months				
Absolute		77.7 (13.4)		78.6 (14.0)
	123		125	
Change from baseline		−1.3 (6.7)		−1.8 (9.7)
% Change from baseline		−1.6 (8.7)		−2.0 (12.4)
48 months				
Absolute		77.6 (12.7)		79.9 (12.0)
	127		124	
Change from baseline		−1.4 (6.6)		−1.5 (5.6)
% Change from baseline		−1.6 (7.9)		−1.7 (6.9)
60 months				
Absolute		77.6 (13.7)		79.7 (12.4)
	116		125	
Change from baseline		−1.7 (7.0)		−1.7 (5.8)
% Change from baseline		−2.2 (8.7)		−1.9 (7.1)
72 months				
Absolute		77.2 (13.7)		79.8 (13.7)
	92			97
Change from baseline		−1.5 (7.0)		−2.3 (6.8)
% Change from baseline		−1.9 (8.6)		−2.6 (7.8)
84 months				
Absolute		76.9 (15.0)		78.1 (11.7)
	90			98
Change from baseline		−1.9 (8.8)		−2.0 (6.8)
% Change from baseline		−2.4 (10.9)		−2.2 (8.4)
96 months				
Absolute		75.8 (13.9)		79.6 (12.3)
	87		95	
Change from baseline		−2.3 (8.6)		−0.7 (9.7)
% Change from baseline		−2.8 (10.8)		−0.3 (14.2)

*SD* standard deviation.

### Disease-free survival

**Fig. 2:**
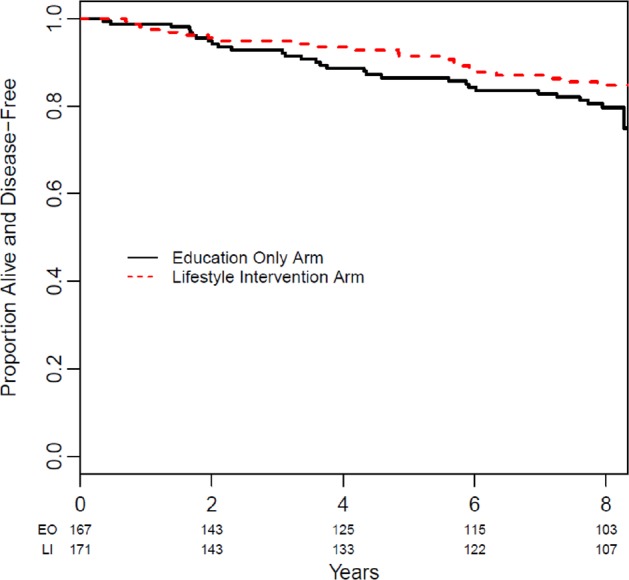
Disease-free survival. Disease free survival in women randomized to the lifestyle intervention arm (dashed red line) and the education only arm (solid black line).

In the lifestyle intervention arm, there were 22 (12.9%) DFS events versus 30 (18.0%) DFS events in the education only arm. There were seven distant recurrences in the lifestyle intervention arm versus 12 in the education only arm; most common sites were bone, liver, and lung. There were nine new primaries in the lifestyle intervention arm and 11 in the education only arm. The most common sites were skin and thyroid. DFS curves by study arm separated at 2 years and remained separate over subsequent follow-up (see Fig. 2). In Cox proportional hazards regression (adjusted for stratification factors), the hazard ratio [HR] for DFS was 0.71, 95% confidence interval [CI]: 0.41–1.24, *p* = 0.23 comparing women in the lifestyle intervention arm to the education only arm. In a landmark analysis, including only those patients (*n* *=* 262, 77.5% of all subjects) who were disease-free at 24 months and who had weight information collected at 24 months, weight change during the first 24 months after randomization was not associated with subsequent DFS (HR per kg weight loss: 0.98, 95% CI: 0.93–1.04, *p* *=* 0.58). This translates to a HR 0.945 (95% CI 0.74–1.20) per 5% weight loss.

### Overall survival

Nine patients in the lifestyle intervention arm (5.3%) and 10 patients in the education only arm (6.0%) died during follow-up (HR: 0.86, 95% CI: 0.35–2.14, *p* *=* 0.74). When the above landmark analysis was performed for OS, a 5% weight loss was associated with a HR 0.72, 95% CI 0.47–1.12, *p* = 0.15). In the lifestyle intervention arm, four of these deaths were related to BC, three to another primary cancer and two to cardiovascular disease. In the education only arm, nine of these events were related to BC, and one to respiratory distress.

### Other outcomes

Other medical events occurred in 146 subjects in the lifestyle intervention arm and 145 in the education only arm. Musculoskeletal events occurred in 106 women in the lifestyle intervention arm and 101 in the education only arm. Cardiovascular, respiratory and gastrointestinal events occurred in 23, 28, and 43 women, respectively, in the lifestyle intervention arm and in 29, 39, and 45 women, respectively, in the education only arm. Osteoporosis was diagnosed in 26 women in the lifestyle intervention arm and 21 women in the education only arm.

## Discussion

Although originally designed as a definitive Phase III adjuvant trial testing the effect of a lifestyle-based weight loss intervention on DFS and OS, accrual to this study was terminated due to loss of funding after 338 women had been enrolled, reducing the power of the study. Despite the inclusion of women with a BMI as low as 24 kg/m^2^ (included with a goal to prevent weight gain), our intervention effectively promoted weight loss—a differential of ~5% favoring the lifestyle intervention arm was seen at 6 and 12 months; however, this differential decreased to 3.3% at 24 months and disappeared by 84 months. This loss of a weight differential between the study arms may have contributed to the lack of association of weight loss at 24 months with subsequent DFS seen in our landmark analysis. The failure of women in the lifestyle intervention arm to maintain weight loss may reflect the fact that the weight loss intervention we used involved only 19 patient contacts over 2 years, fewer than currently considered optimal.^6,8^ Interventions with a greater focus on maintenance of weight loss and relapse prevention would be expected to be associated with less regain of lost weight. More intensive interventions may also lead to greater initial weight loss, which may have larger effects on BC outcomes.

Despite the failure of subjects randomized to our lifestyle intervention to maintain weight loss over time, there were numerically fewer DFS events in women randomized to the intervention arm, with a final point estimate of HR of 0.71 (95% CI: 0.41–1.24, *p* = 0.23). Of interest, the DFS curves separated at 2 years and remained separate (by about 5%) throughout the remainder of the follow-up period. These results are not considered definitive, and should not be used to guide clinical practice, however, we believe they provide important insight into the obesity-breast cancer issue.

There has been considerable discussion in the recent literature regarding the use of *p*-values (or related 95% CIs) alone to interpret research data. The American Statistical Association^10^ has advised against the arbitrary use of a *p*-value of 0.05 to determine whether a treatment effect does or does not exist as this can lead to error, including accepting clinically or scientifically trivial effects as important when *p* < 0.05 and excluding clinically or scientifically important effects when *p* > 0.05. A commentary titled “Retire statistical significance” in Nature^11^ makes a plea for more thoughtful interpretation of research findings, to include an assessment of their potential scientific and clinical importance. They argue that CIs should be considered “compatibility” intervals that provide information on the range of effects sizes that may be plausible.

**Fig. 3:**
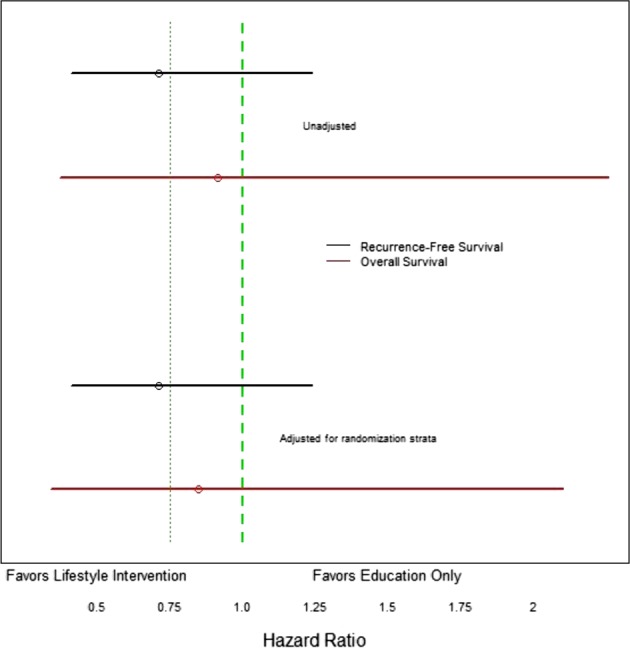
Hazard ratios (HRs) for disease-free survival (DFS), and overall survival (OS). Observed HR for the effect of lifestyle intervention versus education only arm on DFS (black) and OS (red). The upper lines show unadjusted HRs and the lower lines show HRs adjusted for randomization strata (BMI < versus ≥ 30 kg/m^2^, prior chemotherapy, language of intervention). The observed point estimate of the HR is denoted by a circle; the horizontal lines represent 95% CIs. The dotted vertical line represents the hypothesized HR; the dashed vertical line represents no effect.

Applying these perspectives to our findings, our point estimate of the DFS HR for the lifestyle intervention (versus education only) was 0.71—this reflects a potential 29% reduction in risk of recurrence, a reduction that would be clinically important if true. Furthermore, the CIs associated with our HR point estimate are compatible with a beneficial effect on DFS as great as 59% (which would be very important clinically) or an adverse effect as high as 24% (also clinically relevant). Although our results do not provide proof that our intervention improved breast cancer outcomes and should not change clinical practice, it is important to recognize that they are compatible with the beneficial effect of the intervention we had hypothesized (HR 0.75, see Fig. 3). The point estimate is also similar to the HR that would be expected if the adverse effects of obesity that have been identified in meta-analyses (HR 1.3) were reversible with weight loss.^1^ We believe these results add to the expanding literature that weight loss interventions in breast cancer patients may be beneficial. We believe these results serve as additional justification for continuation of ongoing trials, including the Breast Cancer Weight Loss (BWEL) study being conducted by the Alliance,^12^ which should provide robust information regarding the benefits of lifestyle-based weight loss interventions in women with early stage BC.

Strengths of our study include the use of a randomized design, and a standardized weight loss intervention that led to a weight loss differential between study arms during the 2-year intervention with minimal losses to follow-up. Limitations include low power due to early loss of funding and resulting termination of accrual as well as weight regain after the intervention was completed.

Our results are compatible with, although not proof of, a beneficial effect of a weight loss intervention on outcomes in women with BC. They support the conduct of larger trials of standardized interventions that promote sustained weight loss.

## Methods

### Trial design

A Phase III randomized trial was conducted in the adjuvant BC setting, randomizing women who had received standard breast cancer treatment (1:1) to a lifestyle-based weight loss intervention with educational materials versus educational materials only. Enrolment was discontinued when 338 of 2150 subjects had been enrolled and the protocol was amended to allow completion of the study intervention and follow-up, with analysis of the impact of the intervention on disease-free survival (DFS) and overall survival (OS) planned after all patients had a minimum follow-up of eight years. The study design has been previously reported^9^ and is summarized briefly below.

All procedures performed in studies involving human participants were in accordance with the ethical standards of the institutional and/or national research committee; the primary ethics committee was the Ontario Cancer Research Ethics Board with oversight by the Principal Investigator (PG) at Mount Sinai Hospital, Toronto. Informed consent was obtained from all individual participants included in the study.

### Patient population

Randomization occurred August 2007–December 2009. Subjects were required to be postmenopausal, with Stage I, II, or IIIa, (T1–3, pN0-3, M0) estrogen receptor positive (ER+) and/or progesterone receptor positive (PR+) BC within the previous 15 months, with a body mass index [BMI = weight (kg)/height (m^2^)] between 24 and 40 kg/m^2^. In June 2008 entry criteria were changed to allow women with any BMI over 24 kg/m^2^ diagnosed with BC within the previous 36 months to participate and to exclude patients with N3 disease. Patients were required to be on adjuvant letrozole at entry and to be fluent in English or French. Patients were excluded if they had experienced a BC recurrence, had a life expectancy less than 5 years, were unable to walk at least two blocks (self-reported), had insulin requiring diabetes, or other conditions that precluded participation in the intervention. We excluded patients with a prior history of cancer apart from (i) adequately treated non-melanoma skin cancer, (ii) curatively treated in situ carcinoma of the cervix, or (iii) other solid tumors curatively treated with no evidence of disease for ≥5 years.

Randomization was performed centrally (at the Ontario Clinical Oncology Group) using computer generated sequences stratified for (i) BMI (< versus ≥ 30 kg/m^2^), (ii) prior chemotherapy (yes versus no), and (iii) language of the intervention (English versus French).

### Interventions

The study interventions have been previously described.^9^ Both arms received educational material that addressed healthy diets, physical activity, breast cancer, compliance with therapy, osteoporosis and other general medical issues; women also received a 2-year subscription to the Canadian Health Magazine. The lifestyle intervention goals included (i) 10% weight loss to a BMI not less than 21 kg/m/^2^, (ii) caloric reduction to attain a 500–1000 kcal per day deficit with fat intake of ~20% of calories and (iii) a gradual increase in moderate intensity aerobic physical activity (usually walking) to 150–200 min per week, coupled with home-based resistance and stretching exercises. Behavioral components addressed motivation, relapse prevention, emotional distress, time management, and barriers. The intervention was delivered in English or French by trained lifestyle coaches at the University of Ottawa supervised by RS. Nineteen contacts were delivered over 2 years. Calls lasted 30–60 min and were scripted, semi-structured, and standardized; they were based on a participant workbook. Standardization involved training and supervision, recruitment of a Lead Coach, scripting of telephone calls, and recording and centralized review of at least 50% of phone calls.

### Measurements

Women were weighed in indoor clothing, without shoes, at clinic visits at baseline, 6, 12, 18, and 24 months then annually. Height was obtained at baseline.

### Outcomes

The primary outcome was DFS; events included local and regional recurrences (including chest wall), distant recurrence, new primary breast cancers and death, with time from randomization to an outcome event compared in the primary analysis. Secondary outcomes included OS, as well as weight change, quality of life and a composite “other medical” endpoint (diabetes and hospitalization for cardiac or cerebrovascular events, orthopedic events).

### Statistical analysis

DFS and OS were calculated from the date of randomization. Patients were censored on their last follow-up date. Following the intent-to-treat principle, all tests were performed based on randomized allocation. Descriptive statistics were used to summarize patient characteristics and outcomes. Change in weight from baseline was calculated as the absolute change in kg and the percent change from baseline (absolute change/baseline weight × 100). Similar analyses were performed for physical activity and QOL. Differences in QOL and activity level between intervention arms were tested using the Wilcoxon rank sum test at each follow-up time point with data available. The Kaplan–Meier method was used to estimate time-to-event outcomes (DFS, OS). Cox proportional hazards regression was used to investigate factors potentially prognostic of DFS and OS. All analyses included adjustments for stratification factors. Given the reduced power due to early suspension of accrual, no multivariable analyses were performed. A landmark analysis was performed exploring weight loss up to 24 months as a prognostic factor of DFS, including only the subset of patients alive, disease-free and on-study beyond 24 months. The following supportive analyses were performed: using change in BMI instead of change in weight for the landmark analysis, including site as an additional stratification factor, analysis with no stratification factors, and excluding ineligible patients or those who received the incorrect intervention or no intervention. Results were similar, except when site was included as a stratification (all factors statistically not significant). All tests were two-sided and a *p*-value of 0.05 or less was considered statistically significant, with no *p*-value adjustment for multiple testing.

#### Sample size

At the time of study design, it was assumed that 5-year disease-free survival in the control would be 85% and the HR for patients receiving the 24-month intervention would be 0.75. Assuming a total of 8 years of follow-up, a two-sided, *α* = 0.05, log-rank would have >80% statistical power with 2150 patients accrued.

### Reporting summary

Further information on research design is available in the Nature Research Reporting Summary linked to this article.

## Supplementary information

Reporting Summary

## Data Availability

The data generated and analysed during this study are described in the following data record: 10.6084/m9.figshare.11778267.^13^ The data generated during this study are not publicly available in order to protect patient privacy, but a complete de-identified patient-level dataset will be made available to researchers on request, for the purpose of meta-analysis or a newly-proposed study. Data will be made available following submission of a maximum two-page proposal by the requestor. The Study Steering Committee will review, and if applicable, provide approval of the request. A signed data sharing access agreement will be required between the requesting party and the Ontario Clinical Ontology Group (OCOG). The data sharing access agreement is to be executed through either Hamilton Health Sciences or McMaster University, whoever held the original study agreement and/or study account. The data will be provided as SAS datasets (as a CTP or XPT file). Any other format requests may incur costs to the requestor. Data will be available 12 months after publication by the study principal investigator, of the initial study results. The timeframe may vary based on the journal and internal contractual observations. The relevant timeframe should be adjusted accordingly to be study specific. Data availability will end 4 years after the publication of the initial study results. Data requests should be submitted to the OCOG Director, Dr. Mark Levine, email address: mlevine@mcmaster.ca.
